# Hypoxia induces sorafenib resistance mediated by autophagy via activating FOXO3a in hepatocellular carcinoma

**DOI:** 10.1038/s41419-020-03233-y

**Published:** 2020-11-29

**Authors:** Chao Liang, Zhebin Dong, Xianlei Cai, Jie Shen, Yuan Xu, Miaozun Zhang, Hong Li, Weiming Yu, Wei Chen

**Affiliations:** 1 Department of General Surgery, Ningbo Medical Center Lihuili Hospital, Ningbo, 315040 PR China; 2 Cancer Institute of Integrated Traditional Chinese and Western Medicine, Zhejiang Academy of Traditional Chinese Medicine, Tongde Hospital of Zhejiang Province, Hangzhou, 310012 PR China

**Keywords:** Cancer microenvironment, Cancer therapeutic resistance, Chemotherapy

## Abstract

Sorafenib, a multikinase inhibitor, is considered as the only approved drug to cure the advanced hepatocellular carcinoma (HCC); however, the acquired chemoresistance caused by intratumoral hypoxia through sorafenib long term therapy induces sorafenib inefficacy. We demonstrated here that hypoxia significantly attenuated sensitivity of HCC cells to sorafenib treatment and reduced its proliferation. Autophagy was observed in sorafenib-treated HCC cells in hypoxia, and inhibition of autophagy by 3-MA eliminated hypoxia-induced sorafenib resistance. Further study revealed hypoxia-activated FOXO3a, an important cellular stress transcriptional factor, via inducing its dephosphorylation and nuclear location; and FOXO3a-dependent transcriptive activation of beclin-1 was responsible for hypoxia-induced autophagy in HCC cells. Knockout of FOXO3a inhibited the autophagy induced by sorafenib itself in normoxia and significantly enhanced the cytotoxicity of sorafenib in HCC cells; and it also inhibited the hypoxia-induced autophagy and achieved the same effect in sorafenib sensitivity-enhancement in HCC cells as it in normoxia. Finally, knockout of intratumoral FOXO3a significantly enhanced curative efficacy of sorafenib via inhibition of autophagy in xenograft tumors in nude mice. Collectively, our study suggests that FOXO3a plays a key role in regulating hypoxia-induced autophagy in sorafenib-treated HCC, and FOXO3-targeted therapy may serve as a promising approach to improve clinical prognosis of patients suffering from HCC.

## Introduction

Hepatocellular carcinoma (HCC), the most common type of liver cancer, is still a major medical burden worldwide so far^1,2^. HCC represents the fifth most commonly diagnosed cancer and is often diagnosed at advanced stages with high recurrence and mortality rate^3^. Sorafenib, a multikinase inhibitor which suppresses HCC angiogenesis and proliferation, is considered the only effective systemic drug to cure patients with advanced HCC^4^. Nevertheless, sorafenib resistance continually exists in clinical therapy worldwide, and acquired resistance is often observed within 6 months^5^. Multiple intracellular and extracellular factors were considered accounting for sorafenib resistance in HCC^6,7^; however, the precise underlying mechanism is still not understood and, revealing which undoubtedly will benefit for patients with HCC^8^.

Due to uncontrolled growth of HCC leading to intratumor hypoxia, HCC cells are developed with the capacity of more stemness, chemoresistance, and invasive^9,10^. The interactions between hypoxic microenvironment and HCC cells are considered as a key factor causing sorafenib chemotherapy failure^11^. Previous study mostly focused on the activation of hypoxia-inducible factors (HIFs), especially HIF-1α, in hypoxia induced by sustained sorafenib treatment and in turn, HIF-1α mediated cellular responses favored sorafenib resistance^12^. Combination of inhibitor of HIF-1α and sorafenib were documented as a promising approach to reverse sorafenib resistance; however, it did not reach the goal as expect which was considered caused by activation of the compensatory growth pathways^12,13^. Among these mechanisms, autophagy is considered a common and pivotal cellular response to hypoxia^14^. As an evolutionarily conserved process, autophagy has been characterized as a positive cellular adaptive process to maintain the essential homeostasis during stress and recently considered as an importing mechanism accounting for sorafenib resistance^15,16^. Yet up to recent, there are rare reports showing the role of hypoxia-induced autophagy in the sorafenib resistance in HCC.

FOXO3a, an important member of Forkhead box O subfamily, which acts as a transcription factor participating in various cellular responses under stress^17^. Evidences showed FOXO3a plays a key role in regulating autophagy process and may exert opposing effects under different circumstance^18,19^. Though the evidences are relative abundance about autophagy leading to sorafenib resistance, little is known about the biological role of FOXO3a in regulation of autophagy in sorafenib-treated HCC in hypoxia. mTORC1 can be activated by sorafenib via activating AKT, which is a main upstream regulator of FOXO3a, leading to autophagy in HCC cells^20^. And activation of STAT3, a common FOXO3a phosphorylation kinase, could be inhibited by sorafenib, which releases beclin-1 to forming autophagosome^21^. Hence, FOXO3a may participate in the process of sorafenib resistance induced by autophagy theoretically.

In this study, we investigated the role of autophagy on hypoxia-induced sorafenib resistance in HCC cells and verified the function of FOXO3a on regulating autophagy in HCC cells in vitro and in vivo.

## Materials and methods

### Cell lines and experimental conditions

Different types of the human HCC cell lines (Huh-7, well-differentiated, epithelial phenotypic and mutation in p53; LM-3, high-metastatic, epithelial phenotypic and mutation in p53; SNU-387, poor-differentiated, mesenchymal phenotypic and mutation in p53; SNU-449, poor-differentiated, mesenchymal phenotypic and mutation in p53) were purchased from Shanghai Institute for Biological Science (Shanghai, China). Huh-7 and LM-3 cells were cultured in Dulbecco’s minimal essential medium (DMEM; Gibco, Carlsbad, CA, USA) containing 10% fetal bovine serum (FBS; Gibco) and 1% penicillin/streptomycin. SNU-387 and SNU-449 cells were cultured in RPMI 1640 Medium (1640; Gibco) supplemented with 10% FBS and 1% penicillin/streptomycin. For normoxia, cells were maintained at 37 °C in 5% CO_2_ and 95% air; for hypoxia, cells were maintained at atmosphere containing 0.2% O_2_ and 5% CO_2_ in a hypoxia incubator.

### Drugs and antibodies

Sorafenib was purchased from Selleck (Houston, TX, USA). Total-FOXO3a (t-FOXO3a; 12829S), phospho-FOXO3a (Ser253; 9466S), total-AKT (t-AKT; 4685S), phospho-AKT (Ser473; 4058S), LC-3B (3868S) primary antibodies for western blot and immunofluorescence were obtained from Cell Signaling (Danvers, MA, USA). The p62 (ab109012) primary antibody for western blot and immunohistochemical (IHC) was purchased from Abcam (Cambridge, MA, USA). The β-actin (66009-1-Ig) primary antibody was purchased from ProteinTech (Rosemont, IL, USA). The HRP-conjugated secondary antibodies were purchased from Beijing ZhongShan Biotechnology Company (Beijing, China). Anti-rabbit Alexa Fluor 488 (AF488) secondary antibody was purchased from Invitrogen (Carlsbad, CA, USA).

### HCC cells transfection assay

HCC Cells were transfected with scrambled negative control siRNA (NC-siRNA), FOXO3a-siRNA, FOXO1-siRNA (Cell Signaling) or FOXO3a-ΔDBD (FOXO3a overexpression plasmid without transcriptional activity, mutation in FOXO3a DNA binding domain; purchased from GenePharma, Shanghai, China) using Lipofectamine 2000 (Invitrogen). After 6 h transfection, the medium (Opti-MEM; Gibco) was replaced with complete culture medium, and then the transfected HCC cells were incubated in different groups for indicated time periods. All experiments were performed in 72 h after transfection.

### Cell viability and proliferation assay

Cell viability was measured using cell count kit-8 (CCK-8; Dojindo, Kumamoto, Japan) following the manufacturer’s protocol. HCC cells were plated into 96-well plates with conditioned media containing different concentrations of sorafenib for indicated time periods. After incubation with CCK-8 solution per well for 3 h, the absorbance of the wells was measured at 450 nm using an MRX II microplatereader (Dynex, Chantilly, VA, USA). Relative cell viability was calculated as a percentage of untreated control HCC cells. Cell proliferation assay was performed using Click-iT 5-ethynyl-20-deoxyuridine (EdU) Imaging Kit (Invitrogen) as described before^22^.

### Western blots assay

HCC cells lysates were resuspended in cell lysis buffer (Cell Signaling) and the protein concentrations were quantified using BCA Protein assay kit (Thermo Fisher Scientific Inc., Rockford, IL, USA). The prepared protein lysates added with loading buffer and denatured by boiling were separated in 10% SDS-PAGE gels, and then transferred to Polyvinylidene Fluoride (PVDF) membranes (Millipore, Billerica, MA, USA). Then PVDF membranes were incubated with relevant primary antibodies at 4 °C overnight. Then the membranes were incubated with the appropriate HRP-conjugated secondary antibody for 2 h. Protein bands were developed by chemiluminescence (GE Healthcare, Piscataway, NJ, USA) and visualized using an autoradiography kit (Kodak, Rochester, NY, USA).

### Immunofluorescence assay

HCC cells cultured in class bottom cell culture dish were fixed with 4% paraformaldehyde (PFA) before being permeabilized in 0.5% Triton X-100. After blocked with 5% bovine serum albumin (BSA), the HCC cells were incubated with anti-FOXO3a primary antibody at 4 °C overnight and then incubated with anti-rabbit AF488 secondary antibody. The Hoechst 33342 was used for nuclear counterstain. The immunofluorescent images were observed and captured using a confocal laser scanning microscope (Leica, Wetzlar, Germany).

### Detection of autophagic flux

HCC cells were transfected with adenovirus-mRFP-GFP-LC3 to label LC-3. Due to sensitivity to acidic microenvironment, the GFP fluorescence vanish in autolysosome where mRFP retain its red fluorescence. Hence in merged image, the red fluorescence indicates autolysosomes and the merged yellow fluorescence indicates autophagosomes. The change of number of LC-3 fluorescent nots intuitively reflect the autophagic flux level of cells. The transfected HCC were fixed with 4% PFA and observed using a confocal laser scanning microscope. The number of red and yellow fluorescence dots was calculated by manual counting in at least five images of each group.

### Transmission electron microscopy (TEM) assay

For preparation of cell samples observed by TEM, HCC cells were harvested and precipitated by centrifuging. The supernate was removed immediately and the cells were fixed by glutaraldehyde and osmium tetroxide; then the mounted cells were dehydrated using acetone with different concentration successively and embedded in acetone-EPON812. After ultrathin section, the prepared samples were stained by uranyl acetate-lead citrate and observed by an H700 transmission electron microscope (Hitachi, Tokyo, Japan)

### Chromatin immunoprecipitation (ChIP) assay

ChIP assays were performed using kit from Millipore (Darmstadt, Germany) following the manufacturer’s instructions. In brief, HCC cells were fixed by 1% formaldehyde and resuspended by ChIP lysis buffer. The cell lysate was incubated with FOXO3a antibody and the FOXO3a-target gene complex was purified by addition of protein G beads. After elution and decrosslinking, the target gene DNA was collected and quantified by RT-PCR using ABI 7900 Prism HT (Applied Biosystems Inc., Shanghai, China) with indicated gene primers.

### Luciferase reporter assay

The luciferase reporter constructs driven by FOXO3a binding element of beclin-1 promotor or its mutant form (GenePharma) were transfected into LM-3 cells. Then, transfected LM-3 cells were incubated in normoxia or in hypoxia for 48 h, the RNA was isolated and reverse transcribed using Prime Script Reagent RT Kit (Takara, Dalian, China) following the manufacturer’s instructions. The quantification of luciferase mRNA was exerted by RT-PCR using luciferase primers.

### Immunohistochemical assay

The tissue samples from xenograft tumors were fixed in 4% PFA and then sectioned into thin slices. The prepared sections were deparaffinized and rehydrated, and H_2_O_2_ solution was used to suppress the endogenous peroxidase activity. After blocked in 5% BSA, the sections were incubated with p62 primary antibody overnight and then incubated with secondary antibody for 16 h. Finally, the sections were observed and photographed using a digitalized microscope (Nikon, Tokyo, Japan).

### Subcutaneous mouse xenograft of LM-3 cells

Animal researches were executed in compliance with the Guide for the Care and Use of the Animal Ethics Committee of Ningbo University. Three- to four-week-old male nude mice were purchased from Shanghai Experiment Animal Center (Shanghai, China). Prepared LM-3 cells (1 × 10^6^) were injected subcutaneously into the right axillary fossa of each mouse, respectively. Sorafenib (60 mg/kg) therapy was initiated when tumor volumes reached 50–100 mm^3^ and delivered intragastrically every 2 days for 2 weeks. At least 5 nude mice were randomly allocated to each group. Tumor length (*L*) and width (*W*) were measured by sliding caliper every 2 days, and tumor volumes were calculated according to the formula (*L* × *W*^2^)/2^23^. For knockout of FOXO3a, the lentivirus-FOXO3a shRNA was injected into tumor once a week. After 2 weeks of treatment, mice were euthanized by cervical dislocation, then tumors were dissected from each mouse and prepared for subsequent experiments.

### Statistical analyses

All the data were obtained from experiments with adequate sample size and presented as the mean and standard deviation (SD) values. Statistical analysis was performed using Prism 8 (GraphPad, SanDiego, CA, USA). Two-way ANOVA and the two-tailed unpaired Student’s *t*-test were used to assess the significance of different treatments; statistical significance was defined as *p* value <0.05. Each treatment was assayed in triplicate at least (*N* ≥ 3).

## Results

### Hypoxia enhances sorafenib chemoresistance in HCC cells

**Fig. 1:**
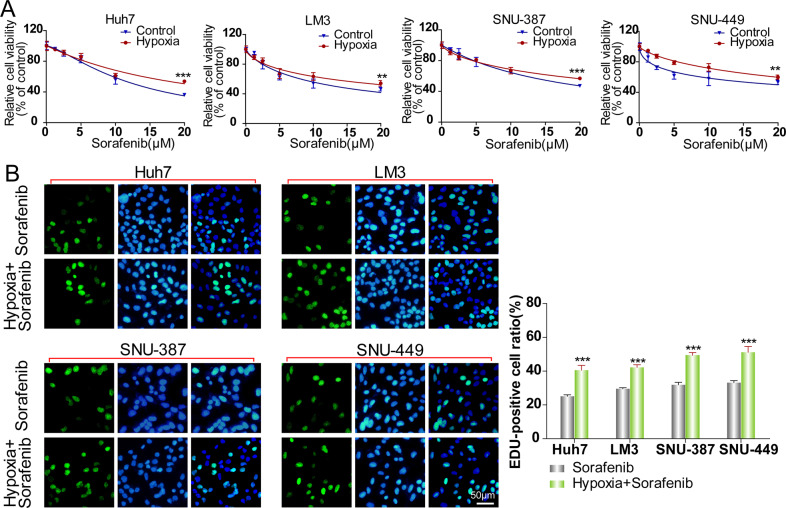
Hypoxia enhances sorafenib chemoresistance in HCC cells. **A** Detection of relative cell viability of HCC cells (Huh-7, LM-3, SNU-387, and SNU-449) cultured in normoxia and hypoxia (Control vs. Hypoxia, ***p < 0.001, **p < 0.01; two-way ANOVA). **B** Detection of proliferative viability of sorafenib-treated HCC cells (Huh-7, LM-3, SNU-387, and SNU-449) cultured in normoxia and hypoxia (Sorafenib vs. Sorafenib+Hypoxia, ***p < 0.001; *t*-test). Each experiment was performed in quintuplicate (*N* = 5). The data are presented as the means ± SD.

The cell viability and proliferative capacity of sorafenib-treated HCC cells in normoxia and hypoxia were tested. After 48 h culture in sorafenib with different concentration, the relative cell viabilities of 4 HCC cells in hypoxia were significantly higher compared to group in normoxia (Fig. 1A). For cell proliferation assay, 4 HCC cells in hypoxia exhibited higher ratio of new DNA synthesis reflected by calculating the Edu-stained-positive cell ratio after 48 h culture with 15 μM sorafenib compared to those in normoxia (Fig. 1B). These results indicated that hypoxia attenuated the cytotoxicity of sorafenib to HCC cells in vitro.

### Hypoxia induces autophagy in HCC cells and inhibition of autophagy significantly enhances cytotoxicity of sorafenib to HCC cells in normoxia and hypoxia

**Fig. 2:**
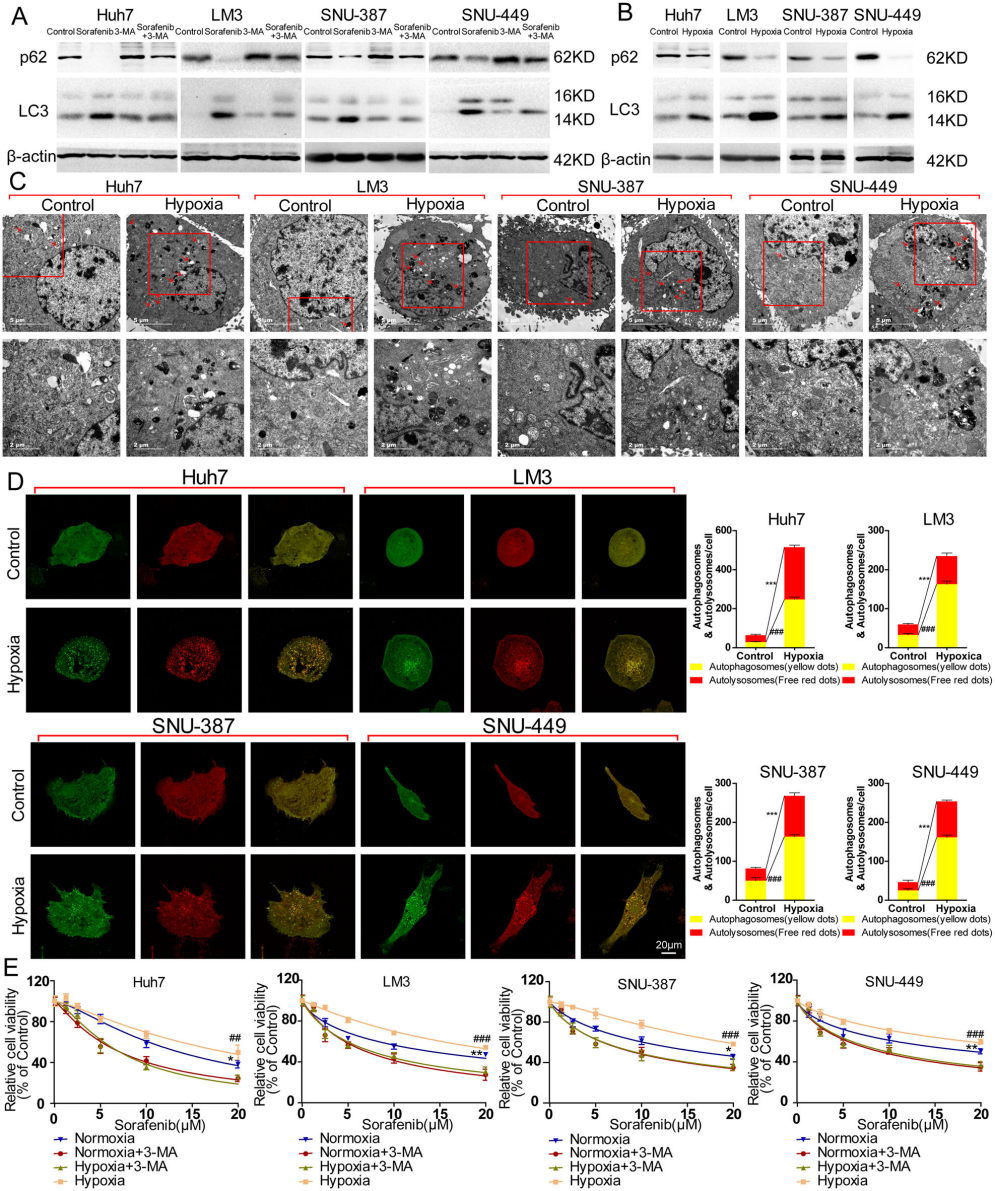
Hypoxia induces autophagy in HCC cells and inhibition of autophagy significantly enhances cytotoxicity of sorafenib to HCC cells in normoxia and hypoxia. **A** Western blot analysis of p62 and LC-3 in HCC cells (Huh-7, LM-3, SNU-387, and SNU-449) cultured with sorafenib alone, 3-MA alone and sorafenib plus 3-MA. **B** Western blot analysis of p62 and LC-3 in HCC cells (Huh-7, LM-3, SNU-387, and SNU-449) cultured in normoxia and hypoxia. **C** Transmission electron microscope image patterns of autophagosome (marked by red arrows; the lower panels are the amplified images of the red frames of upper panels) of HCC cells (Huh-7, LM-3, SNU-387, and SNU-449) cultured in normoxia and hypoxia. **D** Representative images of autophagic flux detected by fluorescence microscope and mean number of yellow dots (autophagosome) and free red dots (autolysosome) per HCC cells (Huh-7, LM-3, SNU-387, and SNU-449) cultured in normoxia and hypoxia (Control vs. Hypoxia, ***p < 0.001, ^###^*p* < 0.001; *t*-test). **E** Detection of relative cell viability of HCC cells (Huh-7, LM-3, SNU-387, and SNU-449) cultured with sorafenib and sorafenib plus 3-MA in normaxia or in hypoxia (Normoxia vs. Normoxia+3-MA, ***p < 0.001; Hypoxia vs. Hypoxia+3-MA, ^###^*p* < 0.001; two-way ANOVA). Each experiment was performed in triplicate (*N* = 3). The data are presented as the means ± SD.

The relationship between autophagy and sensitivity of 4 HCC cells to sorafenib was explored. Western blot assay result revealed that expression of autophagy marker LC-3 was significantly increased after 15 μM sorafenib treatment for 48 h in 4 HCC cells compared to control group, accompanied by the significantly decreased in the expression of substrate recognitive factor p62 in autophagy process (Fig. 2A). The result also showed the sorafenib treatment induced the transition of LC-3I (16KD) to LC-3II (14KD) which represents the activation of autophagic flux (Fig. 2A). 3-MA, a common inhibitor of autophagy, revered the change of expression of LC-3 and p62 induced by sorafenib, which indicated it could effectively inhibited sorafenib-induced autophagy (Fig. 2A).

The expression of LC-3 and p62 of 4 HCC cells after 48 h hypoxic culture were first tested, and the result revealed that hypoxia significantly upregulated expression of LC-3 and downregulated expression of p62 compared to normoxia group (Fig. 2B). TEM was then used to observe the autophagosomes after hypoxia treatment and the amount of the autophagosomes of 4 HCC cells in hypoxia was obviously higher compared to those in normoxia (Fig. 2C; red arrows). Activation of autophagic flux induced by hypoxia was detected by evaluating the extent of autophagosomes and autolysosomes accumulation in mRFP-GFP-LC3 labeled HCC cells. The number of yellow nots indicating autophagosomes and free red nots indicating autolysosomes of 4 HCC cells in hypoxia was much higher than those of them in normoxia (Fig. 2D), which suggested the autophagic flux was obviously activated by hypoxia.

Inhibition of autophagy by 3-MA significantly enhanced the cytotoxicity of sorafenib of different concentration after 48 h culture no matter in normoxia or in hypoxia (Fig. 2E). Though higher cell viability was observed in hypoxia than in normoxia in sorafenib-treated HCC cells, 3-MA-treated HCC cells exhibited a similar cell viability curve in normoxia and in hypoxia under sorafenib treatment, which indicated disrupting of autophagy could reached a same level of inhibition of cell viability in sorafenib-treated HCC cells no matter in normoxia or in hypoxia (Fig. 2E).

### Hypoxia induces retention of FOXO3a in nucleus in HCC cells

**Fig. 3:**
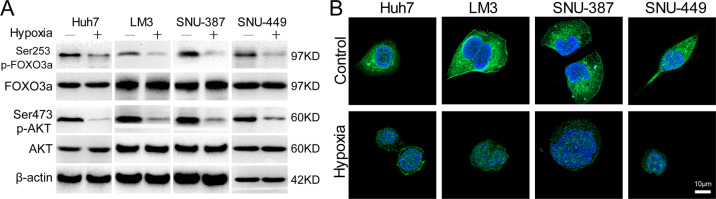
Hypoxia induces retention of FOXO3a in nucleus in HCC cells. **A** Western blot analysis of FOXO3a, AKT, phospho-FOXO3a(Ser253), and phospho-AKT(Ser473) in HCC cells (Huh-7, LM-3, SNU-387, and SNU-449) cultured in hypoxia and normoxia. **B** The subcellular localization of FOXO3a in HCC cells (Huh-7, LM-3, SNU-387, and SNU-449) cultured in hypoxia and normoxia was reflected by immunofluorescence staining patterns of AF-488-labeled FOXO3a. Each experiment was performed in quintuplicate (*N* = 5).

To test the influence of hypoxia in activation of FOXO3a, the phosphorylation and subcellular location of FOXO3a of 4 HCC cells were detected. Compared to normoxia, hypoxia significantly downregulated expression of phosphorylation of FOXO3a (Ser253) and AKT (Ser473) in 4 HCC cells (Fig. 3A). And laser scanning confocal microscope scanning of AF488-labeled FOXO3a revealed that FOXO3a mainly located in nucleus after hypoxia treatment for 48 h, whereas in normoxia, FOXO3a predominantly located in cytoplasm (Fig. 3B).

### FOXO3a plays a critical role in sorafenib-induced autophagy and knockout of FOXO3a significantly enhances the cytotoxicity of sorafenib on HCC cells

**Fig. 4:**
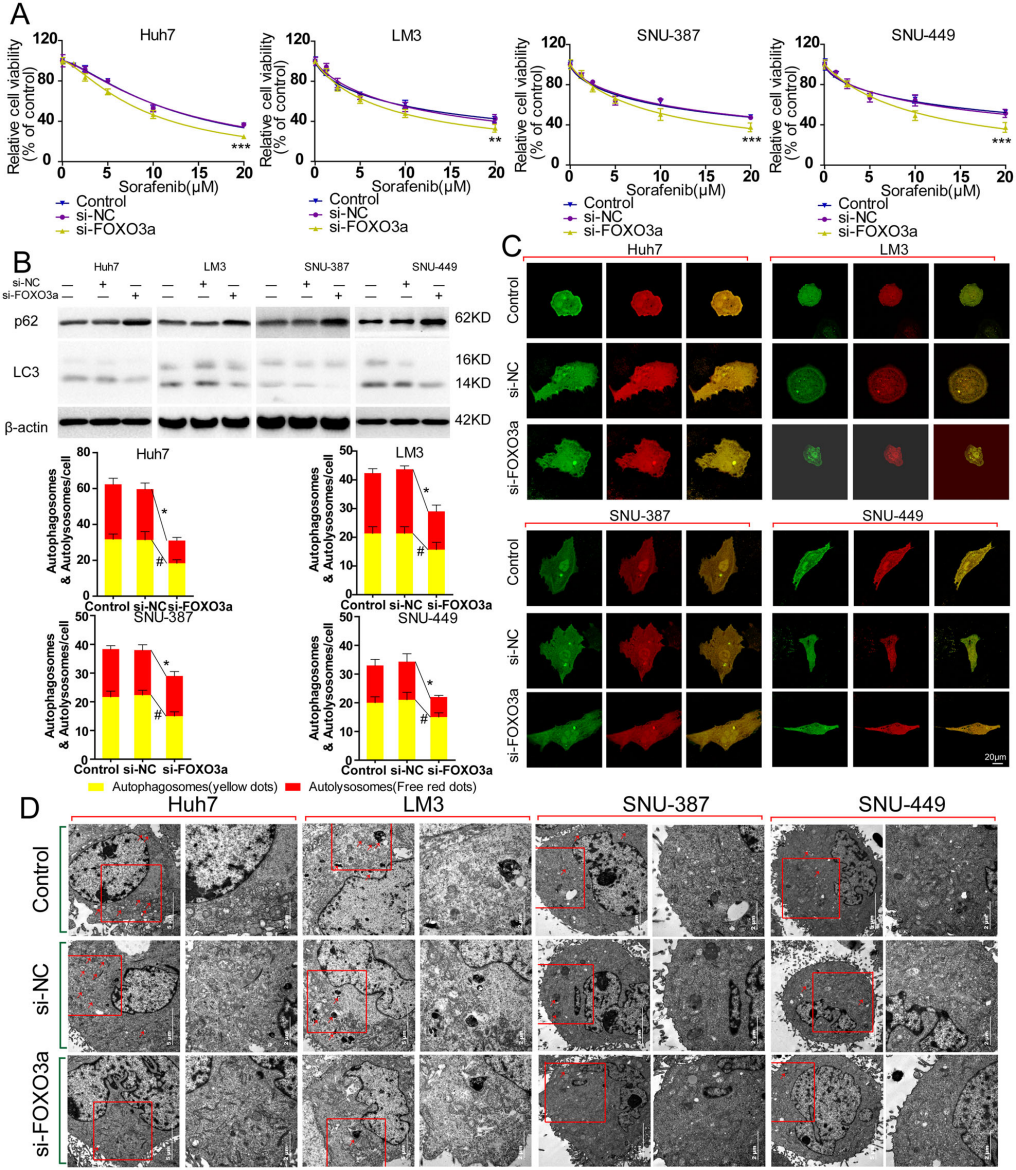
FOXO3a plays a critical role in sorafenib-induced autophagy and knockout of FOXO3a significantly enhances the cytotoxicity of sorafenib to HCC cells. **A** Detection of relative cell viability of sorafenib-treated HCC cells (Huh-7, LM-3, SNU-387, and SNU-449) and sorafenib-treated HCC cells transfected with scramble siRNA (si-NC) and FOXO3a-siRNA (si-FOXO3a; si-NC vs. si-FOXO3a, ***p < 0.001, **p < 0.01; two-way ANOVA). **B** Western blot analysis of p62 and LC-3 in sorafenib-treated HCC cells (Huh-7, LM-3, SNU-387, and SNU-449) and sorafenib-treated HCC cells transfected with si-NC and si-FOXO3a. **C** Representative images of autophagic flux detected by fluorescence microscope and mean number of yellow dots (autophagosome) and free red dots (autolysosome) per sorafenib-treated HCC cells (Huh-7, LM-3, SNU-387, and SNU-449) and sorafenib-treated HCC cells transfected with si-NC and si-FOXO3a (si-NC vs. si-FOXO3a, *p < 0.05, #*p* < 0.05; *t*-test). **D** Transmission electron microscope image patterns of autophagosome (marked by red arrows; the right panels are the amplified image of the red frames of left panels) of sorafenib-treated HCC cells (Huh-7, LM-3, SNU-387, and SNU-449) and sorafenib-treated HCC cells transfected with si-NC and si-FOXO3a. Each experiment was performed in triplicate (*N* = 3). The data are presented as the means ± SD.

To test whether FOXO3a was involved in sorafenib-induced autophagy in HCC cells, FOXO3a was artificiality knockout by siRNA. Cell viability assay result revealed that knockout of FOXO3a significantly enhanced cytotoxicity of sorafenib to 4 HCC cells in normoxia (Fig. 4A). Compared to sorafenib-treated si-NC HCC cells, knockout of FOXO3a obviously upregulated expression of p62 and downregulated expression of LC-3, which meant the activation of autophagic flux induced by sorafenib was blocked by knockout of FOXO3a in 4 HCC cells (Fig. 4B). Observation of autophagosome and autolysosome showed this trend more directly. The number of autolysosomes and autophagosomes in sorafenib-treated si-FOXO3a HCC cells were significantly decreased compared to si-NC group and the merge images showed the decrease in autolysosomes was greater than that in autophagosomes (Fig. 4C). And the amount of autophagosome of sorafenib-treated si-FOXO3a HCC cells observed by TEM was obviously decreased compared to si-NC group (Fig. 4D). These data above suggested that, activation of FOXO3a was necessary for sorafenib resistance induced by autophagy in HCC cells in normoxia.

### Knockout of FOXO3a eliminates hypoxia-induced autophagy and sorafenib resistance in HCC cells

**Fig. 5:**
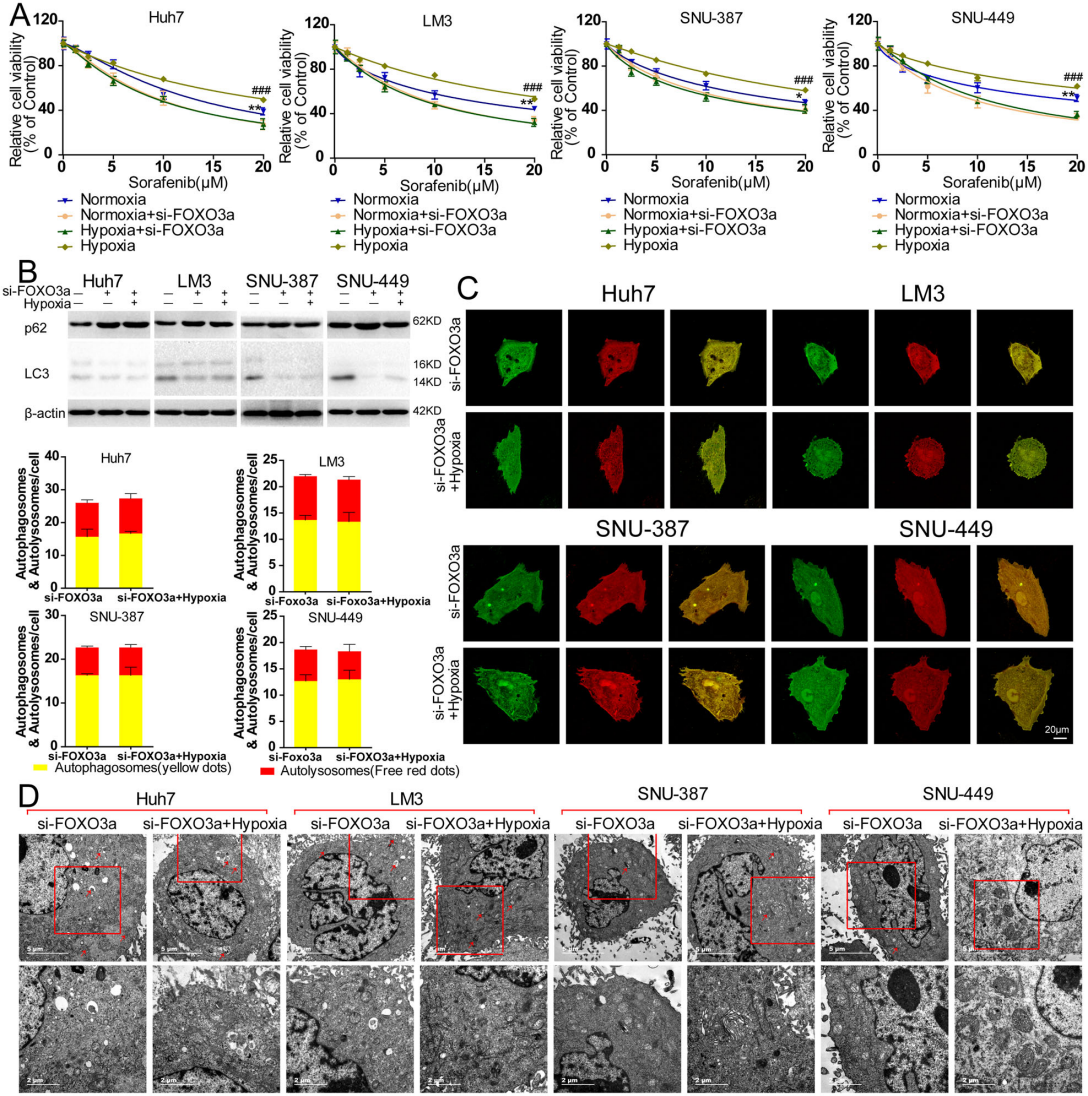
Knockout of FOXO3a eliminates hypoxia-induced autophagy and sorafenib resistance in HCC cells. **A** Detection of relative cell viability of sorafenib-treated HCC cells (Huh-7, LM-3, SNU-387, and SNU-449) and sorafenib-treated HCC cells transfected with FOXO3a-siRNA (si-FOXO3a) cultured in normoxia or in hypoxia (Normoxia vs. Normoxia+si-FOXO3a, *p < 0.05, **p < 0.01; Hypoxia vs. Hypoxia+ si-FOXO3a, ^###^*p* < 0.001; two-way ANOVA). **B** Western blot analysis of p62 and LC-3 in sorafenib-treated HCC cells (Huh-7, LM-3, SNU-387, and SNU-449) and sorafenib-treated HCC cells transfected with si-FOXO3a cultured in normoxia and sorafenib-treated HCC cells transfected with si-FOXO3a cultured in hypoxia. **C** Representative images of autophagic flux detected by fluorescence microscope and mean number of yellow dots (autophagosome) and free red dots (autolysosome) per sorafenib-treated HCC cells (Huh-7, LM-3, SNU-387, and SNU-449) transfected with si-FOXO3a cultured in normoxia and in hypoxia. **D** Transmission electron microscope image patterns of autophagosome (marked by red arrows; the lower panels are the amplified images of the red frames of upper panels) of sorafenib-treated HCC cells (Huh-7, LM-3, SNU-387, and SNU-449) transfected with si-FOXO3a cultured in normoxia and in hypoxia. Each experiment was performed in triplicate (*N* = 3). The data are presented as the means ± SD.

The role of FOXO3a in hypoxia-induced sorafenib resistance was furtherly investigated in HCC cells. Knockout of FOXO3a maintained the cell viability of sorafenib-treated 4 HCC cells in a quiet low level no matter in normoxia or in hypoxia, and showed the similar capacity to enhance the cytotoxicity of sorafenib on HCC cells cultured in hypoxia compared to those in normoxia (Fig. 5A). Western blot analysis showed compared to sorafenib treatment alone, knockout of FOXO3a reversed the upregulation of LC-3 and downregulation of p62 induced by sorafenib treatment (cultured in normoxia) in 4 HCC cells, and it showed the similar trend in sorafenib-treated HCC cells cultured in hypoxia (Fig. 5B). Counting the number of autophagosomes and autolysosomes in sorafenib-treated 4 HCC cells directly showed knockout of FOXO3a blocked the autophagic flux to a same low level no matter in normoxia or in hypoxia (Fig. 5C). And there were no obvious differences in the number of autophagosomes in si-FOXO3a sorafenib-treated HCC in normoxia and in hypoxia observed by TEM (Fig. 5D).

### Transcriptionally activation of beclin-1 by FOXO3a is essential for hypoxia-induced autophagy in HCC cells

**Fig. 6:**
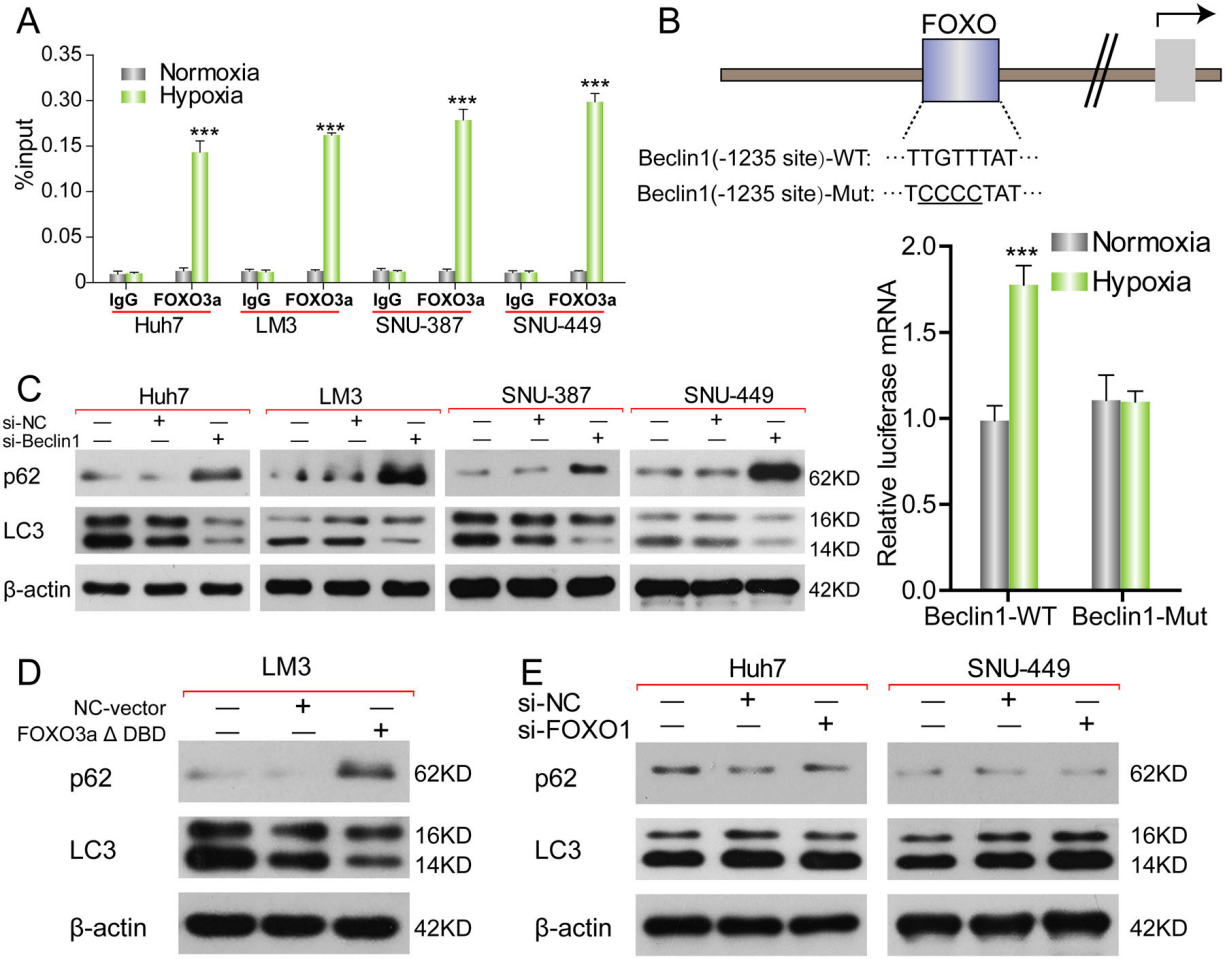
Transcriptionally activation of beclin-1 by FOXO3a is essential for hypoxia-induced autophagy in HCC cells. **A** Detection of binding status of FOXO3a to beclin-1 promotor by ChIP in HCC cells (Huh-7, LM-3, SNU-387, and SNU-449) cultured in normoxia or in hypoxia (Normoxia vs. Hypoxia, ***p < 0.001; *t*-test). **B** Schematic representation of wild-type (WT) beclin-1 promoter and its mutations (MUT). Detection of the relative mRNA level of luciferase in beclin-1 WT LM-3 cells (LM-3 cells transfected with luciferase reporter plasmid containing FOXO3a binding element of beclin-1 promotor) or beclin-1 MUT LM-3 cells (LM-3 cells transfected with luciferase reporter plasmid containing mutant FOXO3a binding element of beclin-1 promotor) cultured in normoxia or in hypoxia by RT-PCR (Normoxia vs. Hypoxia, ***p < 0.001; *t*-test). **C** Western blot analysis of p62 and LC-3 in HCC cells (Huh-7, LM-3, SNU-387, and SNU-449) transfected with si-beclin-1 cultured in hypoxia. **D** Western blot analysis of p62 and LC-3 in LM-3 cells transfected with transfected with FOXO3a-ΔDBD (transcription-inactive FOXO3a plasmid) cultured in hypoxia. **E** Western blot analysis of p62 and LC-3 in HCC cells (Huh-7 and SNU-449) transfected with si-FOXO1 cultured in hypoxia. Each experiment was performed in triplicate (*N* = 3). The data are presented as the means ± SD.

Various autophagy-related genes were reported to be associated with FOXO3a to regulate autophagy^24^. A survey of DNA sequence among the main autophagy-related genes was exerted, and we found that beclin-1, a key regulator of autophagosome formation, contains putative element as FOXO consensus elements. Then we tested if FOXO3a directly bound the beclin-1 promotor locus and if the FOXO3a-dependent transcriptional regulatory was necessary for hypoxia-induced autophagy. ChIP assay results indicated that hypoxia induced binding of FOXO3a to the beclin-1 promotor in 4 HCC cells (Fig. 6A). To further confirm the functionality of the element was crucial for FOXO3a-mediated transcription of beclin-1, we generated the luciferase reporter plasmids of beclin-1 wild-type (WT) promotor and its mutant form (MUT). The luciferase reporter assay revealed that the luciferase mRNA was increased significantly by hypoxia in WT LM-3 cells compared to MUT LM-3 cells (Fig. 6B), demonstrating that FOXO3a binding element in beclin-1 promotor was necessary for FOXO3a-promoted activation of beclin-1 transcription. And knockdown of beclin-1 reversed hypoxia-induced upregulation of LC-3 and downregulation of p62 in 4 HCC cells (Fig. 6C). To furtherly confirm whether the FOXO3a-mediated transcription regulation was essential for hypoxia-induced autophagy, the LM-3 cells were transfected with FOXO3a-ΔDBD and found that hypoxia could not activate the autophagic flux (Fig. 6D). Furthermore, knockdown of FOXO1 by FOXO1-siRNA did not inhibited hypoxia-induced autophagy in Huh-7 and SNU-449 cells (Fig. 6E).

### Knockout of FOXO3a in LM-3 cells subcutaneous xenograft tumors enhances the efficacy of sorafenib in nude mice

**Fig. 7:**
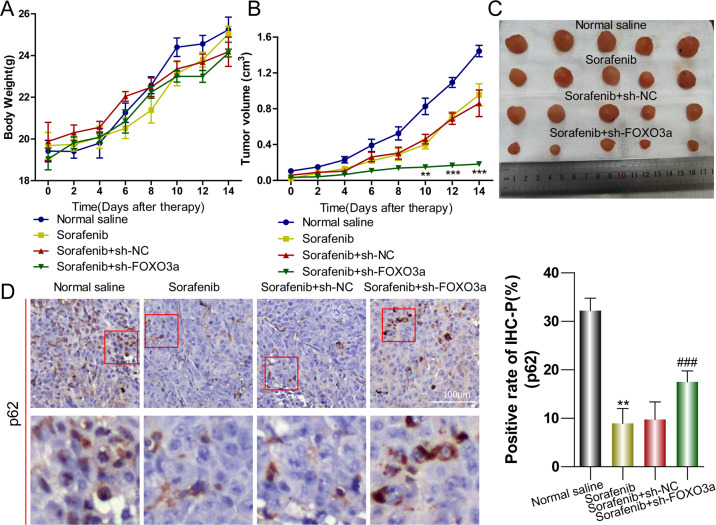
Knockout of FOXO3a in LM-3 cells subcutaneous xenograft tumors enhances the efficacy of sorafenib in nude mice. **A**, **B** Tumor regression rates were calculated by body weight and tumor volume of nude mice with different treatments (cells transfected with scramble shRNA (sh-NC) and FOXO3a shRNA (sh-FOXO3a); Sorafenib+sh-NC vs. Sorafenib+sh-FOXO3a, **p < 0.01, ***p < 0.001; *t*-test). **C** Represen*t*ative pictures of xenograft tumors in nude mice with different treatments after 2 weeks. **D** Immunohistochemical assessment of expression of p62 of xenograft tumors from nude mice with different treatments (the lower panels are the amplified images of the red frames of upper panels; normal saline vs. Sorafenib, **p < 0.01, *t*-test; Sorafenib+sh-NC vs. Sorafenib+sh-FOXO3a, ^###^*p* < 0.001, *t*-test). Each experiment was performed in triplicate (*N* = 3). The data are presented as the means ± SD.

To investigate the role of FOXO3a in sorafenib resistance in vivo, LM-3 subcutaneous xenograft nude mice model was established and the expression of FOXO3a of xenograft tumors was alerted by lentivirus-sh-FOXO3a intratumoral injection. The nude mice body weight did not distinguish in four different treatments groups but intragastric administration of sorafenib obviously reduced the volume of subcutaneous tumors compared to normal saline feeding; and knockout of FOXO3a furtherly reduced tumor volume (Fig. 7A, B). The mice were euthanized after 2 weeks treatment, the subcutaneous xenograft tumors were dissected and analyzed (Fig. 7C). The positive ratio of IHC-p62 of sorafenib therapy alone was significantly lower than that in normal saline feeding, which was reversed by knockout of FOXO3a (Fig. 7D).

## Discussion

More and more researchers found that the extracellular microenvironment of HCC plays an important role in mediating sorafenib resistance^25–27^. As an effective inhibitor of angiogenesis, sustain treatment of sorafenib usually caused intratumoral hypoxia, which in turn promoted activation of HIFs and its downstream pro-survival pathways^28,29^. However, combination of sorafenib and inhibitor of Hif-1α did not exert a promising effort which indicated us there were other mechanisms participating in sorafenib resistance or a deeper functional factor should be investigated. Autophagy is a conservative cell response to various extracellular stress, such as nutrients deprivation, hypoxia, apoptotic stimuli and so on^19^. However, whether hypoxia-induced autophagy mediating sorafenib resistance is still rarely investigated. Liu et al. reported that B-cell lymphoma-2/adenovirus E1B 19 kDa-interacting protein 3 (BNIP3) and BNIP3-like protein X (NIX), the Hif-1α target genes, were activated by hypoxia and induced mitophagy, a specific form of autophagy, which may involve in sorafenib resistance^30^. Wu et al. reported ADRB2-inhibition of autophagy stabling Hif-1α could induce sorafenib resistance^31^. Here our study showed that hypoxia-induced sorafenib resistance could be reversed by 3-MA in HCC cells. Interestingly, inhibition of autophagy induced by hypoxia in sorafenib-treated HCC cells exhibited a similar effort in killing HCC cells compared to those in normoxia. These results indicated us autophagy is a relative major mechanism in mediating hypoxia-induced sorafenib resistance, otherwise inhibition of autophagy of sorafenib-treated HCC cells in hypoxia would not achieve the same effect to those in normoxia if existing other major pro-survival pathways hypoxia could activate. We also found autophagy induced by sorafenib itself was also important in mediating sorafenib resistance, inhibition of sorafenib-induced autophagy significantly enhanced cytotoxicity of sorafenib in HCC cells. Hence, these results indicated us that targeting the autophagy-related pathways may be a good approach to reverse sorafenib resistance in HCC clinical therapy.

FOXO3a functions mainly as transcription factor in regulating cell age, proliferation, apoptosis, stress resistance and metabolism pathways^32–34^. The function of FOXO3a is mainly regulated by its post-translational modification (PTM) of which phosphorylation regulation is the most important and it also decides the subcellular location status of FOXO3a^32^. Phosphorylated FOXO3a binds to protein 14-3-3 which shuttles it to cytoplasm form nucleus which disrupts FOXO3a-dependent transcription^35^. Our team previously reported that FOXO3a participated in regulation of cell proliferation and EMT-induced doxorubicin resistance in HCC^22,36^. However, the association between FOXO3a and sorafenib resistance was rarely investigated until now. Zhao et al. reported activated FOXO3a might facilitate lysosomal proteolysis via autophagy in muscle cells^37^. Recently Wang et al. reported that starvation-induced activation of poly(ADP-ribose) polymerase-1 (PARP1) promoted FOXO3a-depended transcription of autophagy related genes in cardiomyocyte cells and led to autophagy^18^. In this study, we aimed to investigate the role of FOXO3a in autophagy and sorafenib resistance. Idelalisib was reported to reduce AKT signaling and Ser253P-FOXO3a leading to FOXO3a activation and Bim-dependent apoptosis which considered as theoretical basis of combinative treatment of idelalisib and sorafenib in HCC^38^. And sorafenib sustained treatment induced ER stress associated with AMPK-related Ser413P-FOXO3a activation and Bim-dependent apoptosis in HepG2 cells^39^. In acute lymphoblastic leukemia (ALL) cells, sorafenib exerted antiproliferative effect via decreasing AKT signaling and Thr32P-FOXO3a^40^. Our results showed knockout of FOXO3a blocked the autophagy induced by sorafenib and significantly enhanced cytotoxicity of sorafenib in HCC cells. These results demonstrated that FOXO3a-mediated autophagy is an important mechanism in sorafenib resistance in HCC.

HCC cells adapting to intratumorally hypoxia usually exhibited resistance to chemo-agents because of activation of several growth pathways^41,42^. As an important stress factor, FOXO3a was reported involved in hypoxia-induced regulation of cell stress response. Bakker et al. reported FOXO3a was activated by hypoxic stress to inhibit HIF-1-induced apoptosis and promoted cancer cell survive^43^. Jensen et al. reported that FOXO3a acted as HIF-1 downstream to suppress mitochondrial respiration and formation of reactive oxygen species (ROS), and protected cells in hypoxia in vitro and in vivo^44^. We showed here that hypoxia induced Ser253P-FOXO3a dephosphorylation and its nuclear location where FOXO3a exerted its transcriptive function. Knockout of FOXO3a significantly inhibited hypoxia-induced autophagy and reversed sorafenib resistance in vivo and in vitro. And we furtherly confirmed that FOXO3a-dependent promotion of beclin-1 gene transcription was essential for hypoxia-induced activation of autophagic flux in HCC cells. Our results revealed a novel mechanism by which FOXO3a-mediating hypoxia-induced autophagy, as FOXO3a was reported to facilitate autophagy by shuttle FOXO1 to cytoplasm via promoting PI3K transcription^45^. However, our results showed FOXO3a-mediating hypoxia-induced autophagy in HCC cells was not FOXO1 dependent. These results demonstrated that FOXO3a-dependent activation of autophagy-related genes transcription and autophagic flux is a key mechanism mediating hypoxia-induced sorafenib resistance in HCC cells.

The responses to sorafenib varied a lot due to the high heterogeneity of HCC cells, so it is critical to identify a novel and common target to reverse sorafenib resistance. In our study, we selected four HCC cell lines with different differentiation status or phenotype and eventually got the similar results in these cell lines. And these cells harbor different p53 point mutation site and some of them are proved to gain the function facilitating HCC chemotherapeutic resistance. As p53 is also an important factor interacting with autophagy process, it will be worthful to investigate the relationship between p53 status and sorafenib resistance in HCC. Moreover, since sorafenib could not create hypoxic microenvironment in intro, the potential other pathways sorafenib regulated inducing autophagy were also effectively inhibited by FOXO3a, which suggested FOXO3a is a common deep downstream factor in regulating autophagy in sorafenib-treated HCC and targeting FOXO3a could be a more promising approach to reverse clinical sorafenib resistance.

In summary, our founding revealed that hypoxia-induced autophagy is a main mechanism mediating sorafenib resistance in HCC cells and FOXO3a plays a key role in regulating hypoxia-induced autophagy in vitro and in vivo. Therapeutic strategies targeting FOXO3a would be worth exploring to benefit patients with HCC.
